# Dietary Lipid Levels Influence Lipid Deposition in the Liver of Large Yellow Croaker (*Larimichthys crocea*) by Regulating Lipoprotein Receptors, Fatty Acid Uptake and Triacylglycerol Synthesis and Catabolism at the Transcriptional Level

**DOI:** 10.1371/journal.pone.0129937

**Published:** 2015-06-26

**Authors:** Jing Yan, Kai Liao, Tianjiao Wang, Kangsen Mai, Wei Xu, Qinghui Ai

**Affiliations:** 1 The Key Laboratory of Aquaculture Nutrition and Feed (Ministry of Agriculture), Ocean University of China, Qingdao, Shandong province, People’s Republic of China; 2 The Key Laboratory of Mariculture (Ministry of Education), Ocean University of China, Qingdao, Shandong province, People’s Republic of China; East Tennessee State University, UNITED STATES

## Abstract

Ectopic lipid accumulation has been observed in fish fed a high-lipid diet. However, no information is available on the mechanism by which dietary lipid levels comprehensively regulate lipid transport, uptake, synthesis and catabolism in fish. Therefore, the present study aimed to gain further insight into how dietary lipids affect lipid deposition in the liver of large yellow croaker(*Larimichthys crocea*). Fish (150.00±4.95 g) were fed a diet with a low (6%), moderate (12%, the control diet) or high (18%) crude lipid content for 10 weeks. Growth performance, plasma biochemical indexes, lipid contents and gene expression related to lipid deposition, including lipoprotein assembly and clearance, fatty acid uptake and triacylglycerol synthesis and catabolism, were assessed. Growth performance was not significantly affected. However, the hepato-somatic and viscera-somatic indexes as well as plasma triacylglycerol, non-esterified fatty acids and LDL-cholesterol levels were significantly increased in fish fed the high-lipid diet. In the livers of fish fed the high-lipid diet, the expression of genes related to lipoprotein clearance (LDLR) and fatty acid uptake (FABP11) was significantly up-regulated, whereas the expression of genes involved in lipoprotein assembly (apoB100), triacylglycerol synthesis and catabolism (DGAT2, CPT I) was significantly down-regulated compared with fish fed the control diet, and hepatic lipid deposition increased. In fish fed the low-lipid diet, the expression of genes associated with lipoprotein assembly and clearance (apoB100, LDLR, LRP-1), fatty acid uptake (CD36, FATP1, FABP3) and triacylglycerol synthesis (FAS) was significantly increased, whereas the expression of triacylglycerol catabolism related genes (ATGL, CPT I) was reduced compared with fish fed the control diet. However, hepatic lipid content in fish fed the low-lipid diet decreased mainly due to low dietary lipid intake. In summary, findings of this study provide molecular insight into the role of lipid deposition in the liver in response to different dietary lipid contents.

## Introduction

High-lipid diets have increasingly been used for cost-effective farming in aquaculture in recent years. This farming system is based upon the notion that dietary protein can be reduced when more energy is supplied in the form of dietary lipids [1,2]. However, high-lipid diets often lead to ectopic lipid accumulation in the tissues of farmed fish, including the liver and abdominal adipose tissue, and induce metabolic disturbances [3–5]. Lipid deposition is a complex process involving lipid transport, uptake, synthesis and catabolism. In the liver, the lipid stores include fatty acids (FAs) from three sources: diet (delivered *via* chylomicron remnants), *de novo* lipogenesis and circulating non-esterified fatty acids (NEFAs) [6].

Lipid transport in fish is similar to that in mammals, mainly occurring *via* lipoproteins (LPs). The process consists of two loops: an exogenous loop and an endogenous loop [7,8]. The exogenous pathway transports dietary lipids absorbed by the intestine *via* chylomicrons (CMs). The endogenous pathway refers to the hepatic secretion of lipids *via* very low-density LP (VLDL) and the metabolism of VLDL to intermediate-density LP (IDL) and low-density LP (LDL). In mammals, triacylglycerol (TAG) in CMs and VLDL are rapidly hydrolyzed by lipoprotein lipase (LPL) and taken up by peripheral tissues, and the hydrolyzed LP remnants are removed from the plasma *via* LDL receptor (LDLR) and LDL receptor-related protein-1 (LRP-1)mainly by the liver but also partially by peripheral tissues [9–12]. Additionally, scavenger receptor class B type I (SRBI) is also closely related to CM and VLDL metabolism. Although LP receptors play an important role in lipid transport, few studies have specifically targeted LP receptors in fish. Long-chain FAs (LCFAs) released from TAG in LPs and other sources have been widely demonstrated to cross the plasma membrane into different tissues *via* a protein-mediated mechanism [13–15]. In adipocytes, protein-mediated LCFA uptake accounts for approximately 90% of cellular acquisition [16]. A number of membrane FA transporters have been identified, including FA translocase (cluster of differentiation, CD36), a family of FA transport proteins (FATP1-6) and plasma membrane-associated FA binding proteins (FABP_pm_). Subsequently, FAs transported across the membrane are transferred through the cytosol *via* FA binding proteins (FABPs) for oxidation or storage. FABPs are ubiquitously expressed in vertebrate tissues, with distinct expression patterns being observed for individual FABP and are named after the tissues in which they were discovered or are mainly expressed, such as basic liver-type FABP (FABP10), heart-type FABP (FABP3) and adipocyte-type FABP (FABP4) [17]. In fish, FABP10 mRNA is highly expressed in the liver, whereas FABP3 is abundantly expressed in muscle. Although FABP4 has not been described, FABP11 may represent the fish lipid storage tissue FABP homologue [18,19]. It should be noted that most tissues express various FABP types because no FABP is specific to a given tissue. The function and regulation of FA transporters and FABPs have been studied under various nutritional and hormonal conditions in fish [13–15,18,20–22]. However, these studies primarily focused on the regulation of hormonal stimulation. In addition to lipid transport and uptake, TAG synthesis and catabolism also influence lipid deposition, and the regulation of these processes has been extensively demonstrated in fish [23,24].

The large yellow croaker (*Larimichthys crocea*) is a commercially important carnivorous species for marine culture given its high market value in China. To our knowledge, no information is available on the mechanism by which dietary lipid levels comprehensively regulate lipid transport, uptake, synthesis and catabolism in fish. Thus, the aim of this study was to investigate the effect of dietary lipid levels on lipid deposition in the liver of large yellow croaker and to determine the mechanism by which dietary lipids regulate lipid deposition at the transcriptional level.

## Materials and Methods

### Ethics statement

The present study was performed in strict accordance with the Standard Operation Procedures (SOPs) of the Guide for the Use of Experimental Animals of Ocean University of China. All animal care and use procedures were approved by the Institutional Animal Care and Use Committee of Ocean University of China (Permit Number: 20001001). Fish were anesthetized with eugenol (1:10,000) (Shanghai Reagent Corp., Shanghai, China) to minimize suffering before being assigned to cages and sampling.

### Diet and feeding trial

Three isoproteic (42% crude protein) diets were formulated to contain low (6%), moderate (12%) and high (18%) crude lipid levels by gradually adding fish oil, and wheat starch was used to adjust the level of fish oil. The diet with 12% crude lipid was used as the control, which has been proven to be appropriate for the growth of this fish [25]. The formulation and approximate composition of the diets are presented in Table 1, and the FA composition of the diets is shown in Table 2.

**Table 1:** Formulation and proximate composition of the experimental diets.

	Dietary lipid levels (%)		
	Low (6)	Moderate (12)	High (18)
Ingredients (g/100 g)			
Fish meal^1^	39.0	39.0	39.0
Soybean meal^1^	20.0	20.0	20.0
Wheat meal^1^	23.3	23.3	23.3
Wheat starch^1^	12.0	6.0	0
Fish oil^1^	0	6.0	12.0
Soybean lecithin^1^	1.5	1.5	1.5
Vitamin premix^2^	2.0	2.0	2.0
Mineral premix^3^	2.0	2.0	2.0
Attractant^4^	0.1	0.1	0.1
Mold inhibitor^5^	0.1	0.1	0.1
Proximate composition (g/100 g)			
Moisture	9.5	9.4	9.2
Crude protein	43.1	42.6	43.2
Crude lipid	6.1	11.5	17.8

^1^All of these ingredients were supplied by Great Seven Biotechnology Co., Ltd., China.

^2^Vitamin premix (mg or g/kg diet): cholecalciferol, 5 mg; retinol acetate, 32 mg; thiamin 25 mg;vitamin B_12_ (1%), 10 mg; riboflavin, 45 mg; pyridoxine HCl, 20 mg; ascorbic acid, 2000 mg; alpha-tocopherol (50%), 240 mg; vitamin K_3_, 10 mg; pantothenic acid, 60 mg; inositol, 800 mg; niacin acid, 200 mg; folic acid, 20 mg; biotin (2%), 60 mg; choline chloride (50%), 4000 mg;microcrystalline cellulose, 12.47 g.

^3^Mineral premix (mg or g/kg diet): CuSO_4_·5H_2_O, 10 mg; Ca (IO_3_)_2_·6H_2_O (1%), 60 mg; CoCl_2_·6H_2_O (1%), 50 mg; FeSO_4_·H_2_O, 80 mg; MgSO_4_·7H_2_O, 1200 mg; MnSO_4_·H_2_O, 45 mg; NaSeSO_3_·5H_2_O (1%), 20 mg; ZnSO_4_·H_2_O, 50 mg; CaH_2_PO_4_·H_2_O, 10 g; zeolite, 8.485 g.

^4^Attractants: glycine and betaine.

^5^Mold inhibitor: contained 50% calcium propionic acid and 50% fumaric acid.

**Table 2:** Fatty acid composition of the experimental diets (for total fatty acids).

		Dietary lipid levels (%)	
Fatty acid (%)	Low (6)	Moderate (12)	High (18)
C14:0	4.90	6.17	6.17
C16:0	20.81	20.43	19.52
C18:0	3.27	2.92	2.51
C20:0	nd^1^	1.72	2.26
ΣSFA^2^	28.97	31.23	30.46
C16:n-9	8.61	8.83	8.68
C18:n-9	14.76	19.18	22.31
C22:n-9	nd	1.21	1.75
ΣMUFA^3^	23.36	29.22	32.74
C18:2n-6	20.46	12.53	9.86
C18:3n-6	0.79	1.43	1.46
C20:4n-6	0.65	0.71	0.65
Σn-6PUFA^4^	21.90	14.67	11.97
C18:3n-3	2.10	1.86	1.71
C18:4n-3	nd	0.31	0.39
C20:5n-3 (EPA)	10.22	9.19	8.25
C22:6n-3 (DHA)	8.20	8.46	8.68
Σn-3PUFA	20.52	19.81	19.03
n-3/n-6PUFA	0.94	1.35	1.59
Σn-3LC-PUFA^5^	18.41	17.65	16.93
DHA/EPA	0.80	0.92	1.05

^1^nd: not detected.

^2^SFA: saturated fatty acids.

^3^MUFA: monounsaturated fatty acids.

^4^PUFA: polyunsaturated fatty acids.

^5^LC-PUFA: long-chain-polyunsaturated fatty acids.

Large yellow croaker of the Tai-chu race were obtained from a local farm (Aquatic Fingerlings Limited Company of Xiangshan Harbour, Ningbo, China) located at [121.752, 29.545]. The fish were reared in floating sea cages (3.0×3.0×3.0 m) for 2 weeks for acclimation to the experimental conditions. Before the feeding trial, fish were fasted for 24 h and weighed after being anaesthetized with eugenol (1:10,000) (Shanghai Reagent Corp., Shanghai, China). Fish of similar sizes with an initial weight of 150.00±4.95 g were randomly assigned to nine cages (1.5×1.5×2.0 m) with 40 fish per cage. Each diet was randomly distributed in triplicate cages. The fish were hand-fed to apparent satiation twice daily (05:00 and 17:00). During the 10-week feeding trail, the water temperature ranged from 21 to 28.5°C. In addition, the salinity ranged from 28 to 32‰ and the dissolved oxygen level ranged from 6.7 to 7.8 mg/L.

### Sample collection

At the end of the trial, the fish were fasted for 24 h and anesthetized with eugenol (1:10,000). Then, the fish in each cage were weighed and counted. Five fish per cage were randomly selected for proximate analysis. Blood was sampled from the caudal veins of four fish per cage using ethylenediaminetetraacetic acid(EDTA)-containing Vacutainers (Huabo medical instrument Co., Ltd, Heze, Shandong province, China). Plasma was separated from the blood *via* centrifugation at 3000 rpm for 10 min at 4°Cand stored at -80°C until use. After blood sampling, the fish were dissected to calculate the hepato-somatic index (HSI) and viscera-somatic index (VSI). The liver was sampled for moisture and lipid contents analyse. For molecular analysis, the livers of three fish per cage were sampled and immediately transferred to liquid nitrogen and then stored at -80°C until analysis.

### Plasma biochemical analysis

The plasma samples from each cage were pooled. Plasma TAG, total cholesterol, LDL-cholesterol (LDL-c) and high-density lipoprotein-cholesterol (HDL-c) concentrations were analyzed using commercial assay kits (Mindray Bio Medical Co., Ltd., Shenzhen, China)and a Mindray Auto Bio-chemical Analyzer (BS-400, Mindray, Shenzhen, China) according to the manufacturer’s instructions. TAG was measured *via* the glycerol lipase oxidase (GPO-PAP) method[26]. Total cholesterol, LDL-c and HDL-c were determined using the cholesterol oxidase method based on Schettler and Nussel [27], Okada *et al*. [28] and Gordon *et al*. [29], respectively. NEFA levels were measured using a Cu-NEFA coextraction-based colorimetric assay kit (Nanjing Jiancheng Bioengineering Inc., Nanjing, China) according to Falholt *et al*. [30].

### Measurement of moisture and lipid contents

Whole body moisture content was analyzed by drying the samples to constant weight at 105°C. The moisture contents of the liver were determined *via* the freeze-drying method using vacuum-freezing drying equipment (Christ ALPHA, Germany). The crude lipid content of the whole body was measured through ether extraction using the Soxhlet method (Soxhlet Extraction System B-811, Switzerland). Approximately 100 mg of dried liver tissue was subjected to lipid extraction *via* the Folch method [31]. Briefly, the tissues were first homogenized with 6 mL of chloroform-methanol (2:1) and then centrifuged at 3000 rpm for 15 min to recover the liquid phase. The upper solvent was washed with 1.2 mL of a 1.6% CaCl_2_ solution. The lower chloroform phase containing lipids was first dried under nitrogen and then dried to constant weight at 75°C.

### RNA extraction and real-time quantitative PCR

Total RNA was extracted from the liver using TRIzol reagent (Invitrogen, Carlsbad, CA,USA). Isolated RNA quantity and quality were determined *via* spectrophotometry using a NanoDrop spectrophotometer and on a 1.2% denaturing agarose gel, respectively. For cDNA synthesis, the TransScript One-step gDNA Removal and cDNA Synthesis Super Mix Kit (Transgen Biotech, Beijing, China) were used according to the manufacturer’s instructions. cDNA was diluted 5-fold using RNase- and DNase-free water. Real-time quantitative PCR was performed in a quantitative thermal cycler (Mastercycle ep realplex, Eppendorf, Hamburg, Germany). PCR measurements were performed in a total volume of 20μL, containing 0.4μL of each primer (10μM), 10μL of 2×TransStart Top green qPCR SuperMix (Transgen Biotech, Beijing, China) and 0.8 μL of cDNA. The following quantitative PCR program was employed: 95°C for 2 min, followed by 40 cycles of 95°C for 10 s, 60°C for 10 s and 72°C for 20 s. The primer sequences for each gene are listed in Table 3. At the end of PCR amplification, melting curve analysis was performed to confirm that only one PCR product was present. The fluorescence data acquired during the extension phase were normalized to β-actin *via* the delta-delta method [32]. Normalized gene expression for the control diet group (12% dietary lipid) was set at 1.

**Table 3:** Primer pair sequences and amplicon size of the genes used for real-time PCR.

	5′-3′ primer sequence			
Gene	Forward	Reverse	Amplicon size (bp)	GenBank accession no.
apoAI	ttgctctcgcccttctcctg	cacgctgtccttgatctccttg	179	KM593125
apoB100	agagtgttgtccaggataaagatgc	cagggctcagggtctcagtc	147	KM593126
MTP	atgtccaaaatgttctccatgtctg	atgtcaatagccaaccctccttg	143	KP027412
LPL	gaattcaacgcggaaacacag	acgctcatagagggcagacac	105	JQ327827
LDLR	acataagcgccggtgctgtt	tacgatgtcctctggctgattc	95	KM593127
LRP-1	tggactgggtggctggaaac	caatggcgtatggctcgtctatc	126	KM593128
SRBI	acagatccagaaagacaacatcacg	gtagggcaacttctccatcatcac	172	KM593129
CD36	gagcatgatggaaaatggttcaaag	ctccagaaactccctttcaccttag	159	KM593122
FATP1	caaccagcaggacccattacg	catccatcaccagcacatcacc	131	KM593124
FABP3	ccaaacccaccactatcatctcag	gcaccatctttccctcctctattg	171	KM593123
FABP10	caatggaacatggcaggtttacg	tgattggcttgatgtccttggc	107	KM593131
FABP11	caggtgggcaatcggaccaa	ggctcgttgagcttgaacttga	119	KM593130
FAS	cagccacagtgaggtcatcc	tgaggacattgagccagacac	126	JX456351
DGAT2	ttcggtgctttctgcaacttcg	aaggatggggaagcggaagt	111	KJ563922
ATGL	ccatgcatccgtccttcaacc	Gagatccctaaccgcccact	103	HQ916211
CPT I	gctgagcctggtgaagatgttc	Tccatttggttgaattgtttactgtcc	159	JX434612
ACO	agtgcccagatgatcttgaagc	Ctgccagaggtaaccatttcct	184	JX456348
β-actin	ctacgagggttatgccctgcc	Tgaaggagtaaccgcgctctgt	107	GU584189

apo: apolipoprotein; MTP: microsomal TAG transfer protein; LPL: lipoprotein lipase; LDLR: low-density lipoprotein receptor; LRP-1: lipoprotein receptor-related protein-1; SRBI: scavenger receptor class BI; CD36: cluster of differentiation 36; FATP1: fatty acid transport protein 1; FABP: fatty acid binding protein; FAS: fatty acid synthase; DGAT2: acyl-CoA: diacylglycerol acyltransferase 2; ATGL: adipose triglyceride lipase; CPT I: carnitine palmitoyltransferase I; ACO: acyl-CoA oxidase.

### Statistical analysis

Statistical analysis was performed using SPSS 13.0 for Windows (SPSS Inc., Chicago, IL, USA). The data are presented as means with the standard error of the means (S.E.M.). The results were analyzed *via* one-way analysis of variance (ANOVA) followed by Tukey’s multiple-range test. When the homogeneity of variance was not satisfied, an independent-samples t test was performed to compare the differences. The level of significance was set at *P*<0.05.

## Results

### Survival rate, growth performance and somatic and plasma biochemical indexes

The survival rate, final weight, specific growth rate, feed intake and feed conversion ratio of the fish were not significantly affected by dietary lipid levels (*P*>0.05) (Table 4). Both HSI and VSI increased with increasing dietary lipid content. In particular, fish fed the high-lipid diet exhibited significantly increased HSI and VSI compared with fish fed the low-lipid and control diets (*P*<0.05) (Table 4). Plasma TAG, NEFA, and LDL-c levels significantly increased as the dietary lipid content increased from 6 to 18% (*P*<0.05), whereas no significant difference in plasma total cholesterol and HDL-c levels was observed among treatments (*P*>0.05).

**Table 4:** Effects of dietary lipid levels on the growth performance, somatic parameters and plasma biochemical indexes of large yellow croaker (*Larimichthys crocea*)*.

		Dietary lipid levels (%)	
	Low (6)	Moderate (12)	High (18)
Survival rate (%)^1^	81.58±2.63	93.86±3.16	85.96±3.16
Final weight (g)	281.55±4.47	280.17±3.85	269.3±3.13
Specific growth rate (%day^-1^)^2^	0.79±0.02	0.85±0.02	0.77±0.02
Feed intake^3^	0.57±0.02	0.55±0.01	0.54±0.01
Feed conversion ratio^4^	1.91±0.08	1.64±0.07	1.80±0.09
Hepato-somatic index^5^	1.64±0.05^b^	1.82±0.04^b^	2.05±0.06^a^
Viscera-somatic index^6^	6.73±0.12^b^	7.37±0.16^b^	8.17±0.19^a^
Total cholesterol (mmol/L)	2.38±0.28	2.88±0.36	3.08±0.47
Triacylglycerol (mmol/L)	2.77±0.11^c^	4.55±0.03^b^	5.85±0.36^a^
NEFA (μmol/L)^7^	96.57±12.97^b^	123.57±8.31^ab^	154.72±5.49^a^
HDL-cholesterol (mmol/L)	0.40±0.05	0.42±0.06	0.38±0.05
LDL-cholesterol (mmol/L)	0.13±0.02^b^	0.21±0.03^b^	0.39±0.03^a^

*Values (means±S.E.M.) that share the same letter in the same row are not significantly different (*P*<0.05; Tukey’s test) among treatments (n = 3).

^1^Survival rate (%) = 100×final fish number/initial fish number.

^2^Specific growth rate (%day^-1^) = 100×[ln (final weight)-ln (initial weight)]/days.

^3^Feed intake (day^-1^) = feed consumption (g)/(days×(final body weight + initial body weight)/2).

^4^Feed conversion ratio = feed consumption (g)/wet weight gain (g).

^5^Hepato-somatic index = 100×(liver weight/body weight).

^6^Viscera-somatic index = 100×(viscera weight/body weight).

^7^NEFA: non-esterified fatty acids.

### Moisture and lipid contents

The whole body and liver moisture contents decreased as dietary lipids increased, and these levels were significantly reduced in fish fed the high-lipid diet compared to fish fed the low-lipid diet (*P*<0.05) (Table 5). However, whole body and liver lipid contents were significantly elevated as dietary lipid content increased from 6 to 18% (*P*<0.05). In particular, the liver lipid content determined in fish fed the high-lipid diet (26.9%) was up to 77% increased compared with fish fed the low-lipid diet (15.1%) in terms of wet weight.

**Table 5:** Effects of dietary lipid levels on the moisture and lipid contents of the whole body, liver and muscle of large yellow croaker (*Larimichthys crocea*) (%, wet weight)*.

		Dietary lipid levels (%)	
	Low (6)	Moderate (12)	High (18)
Whole body (g/100 g)			
Moisture	72.36±0.19^a^	68.88±0.51^b^	68.86±0.39^b^
Lipid	9.56±0.12^b^	11.45±0.2^a^	11.83±0.25^a^
Liver (g/100 g)			
Moisture	61.41±0.40^a^	56.90±0.44^b^	55.12±0.31^c^
Lipid	15.14±0.37^c^	23.01±0.26^b^	26.91±0.82^a^

*Values (means±S.E.M.) that share the same letter in the same row are not significantly different (*P*<0.05; Tukey’s test) among treatments (n = 3).

### Expression of proteins related to VLDL assembly in the liver

**Fig 1:**
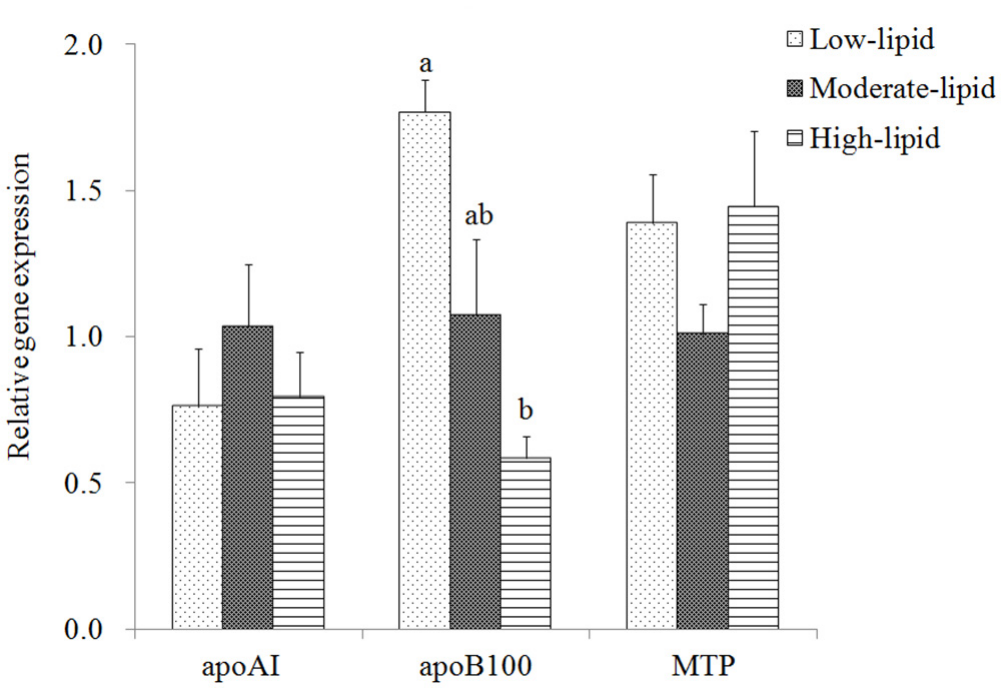
Expression of genes related to VLDL assembly in the liver of large yellow croaker. Values (means±S.E.M.) in bars that have the same letter are not significantly different (*P*>0.05; Tukey’s test) among treatments (n = 3). apo: apolipoprotein; MTP: microsomal triacylglycerol transfer protein.

Among the three proteins (apoAI, apoB100 and MTP) involved in VLDL assembly in the liver, only apoB100 mRNA levels exhibited a significant difference among the three groups (Fig 1). The expression of apoB100 was significantly down-regulated in fish fed the high-lipid diet compared with those fed the low-lipid diet (*P*<0.05).

### Expression of lipoprotein lipase and lipoprotein receptors

**Fig 2:**
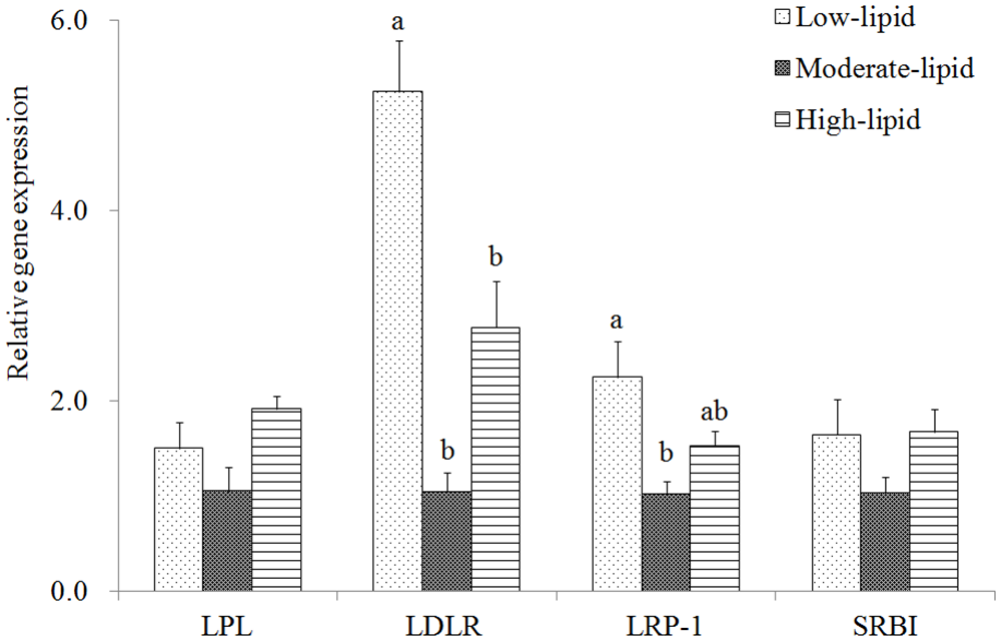
Expression of genes encoding LPL and lipoprotein receptors in the liver of large yellow croaker. Values (means±S.E.M.) in bars that have the same letter are not significantly different (*P*>0.05; Tukey’s) among treatments (n = 3). LPL: lipoprotein lipase; LRP-1: LDL receptor-related protein-1; SRBI: scavenger receptor class BI.

LPL expression did not show any significant changes in the liver among the dietary treatments (*P*>0.05) (Fig 2). The lowest expression of LDLR and LRP-1 was noted in the control group, whereas the highest expression was observed in the low-lipid group, and there were significant differences between these two groups (*P*<0.05). SRBI expression was not significantly affected by dietary lipids (*P*>0.05).

### Expression of genes related to fatty acid uptake

**Fig 3:**
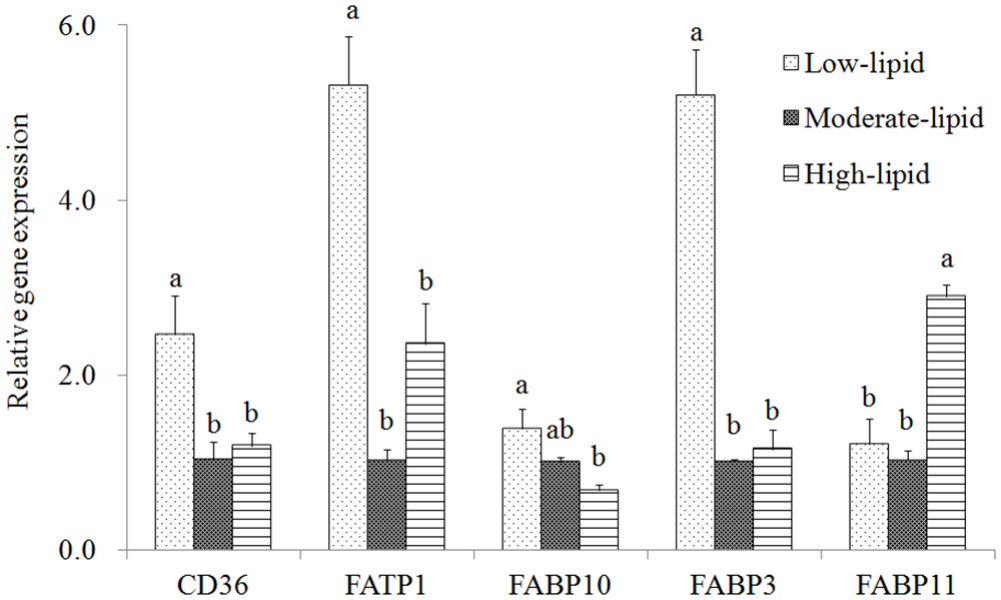
Expression of genes related to fatty acid uptake in the liver of large yellow croaker. Values (means±S.E.M.) in bars that have the same letter are not significantly different (*P*>0.05; T test for FABP3 in the liver, and Tukey’s test for the other genes) among treatments (n = 3). CD36: cluster of differentiation; FATP1: fatty acid transport protein1; FABP: fatty acid binding protein.

Compared with the control group, only FABP11 expression was significantly increased in the high-lipid group (*P*<0.05)(Fig 3). No significant changes in the expression of CD36, FATP1, FABP10 and FABP3 were observed between the high-lipid and control groups (*P*>0.05) though FATP1 was 1.4-fold up-regulated in the high-lipid group. In the low-lipid group, the expression of CD36, FATP1 and FABP3 was significantly up-regulated compared with the control group (*P*<0.05), whereas FABP10 and FABP11 mRNA levels did not significantly differ in these two groups. The expression of all of the investigated genes involved in FA uptake (CD36, FATP1, FABP3 and FABP10)was significantly increased in the low-lipid group compared with the high-lipid group with the exception of FABP11, which was expressed at a higher level in the high-lipid group (*P*<0.05).

### Expression of genes involved in triacylglycerol synthesis and catabolism

**Fig 4:**
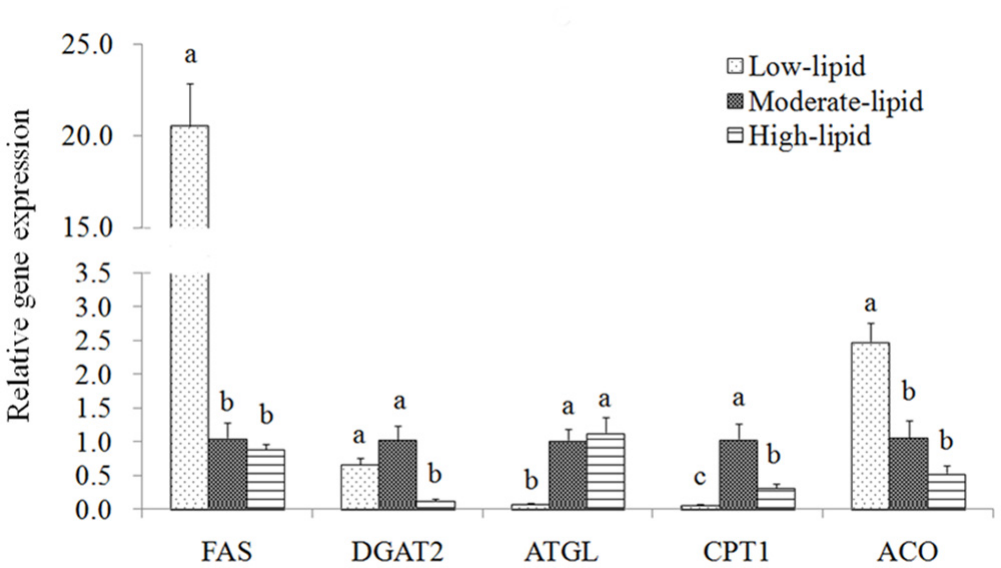
Expression of genes related totriacylglycerol synthesis and catabolism in the liver of large yellow croaker. Values (means±S.E.M.) in bars that have the same letter are not significantly different (*P*>0.05; T test for FAS, DGAT2 and CPT I in the liver, and Tukey’s test for the other genes) among treatments (n = 3). FAS: fatty acid synthase; DGAT2: acyl-CoA:diacylglycerol acyltransferase 2; ATGL: adipose triglyceride lipase; CPT I: carnitine palmitoyltransferase I; ACO: acyl-CoA oxidase.

In fish fed the high-lipid diet, the expression of DGAT2 and CPT I was significantly down-regulated compared with fish fed the control diet (*P*<0.05), whereas the expression of FAS, ATGL and ACO was not significantly altered between fish fed the high-lipid and control diets (*P*>0.05)(Fig 4). In fish fed the low-lipid diet, FAS and ACO were significantly up-regulated, whereas ATGL and CPT I were down-regulated compared with fish fed the control diet (*P*<0.05). TAG synthesis related genes (FAS and DGAT2) were expressed at higher levels in the low-lipid group compared with the high-lipid group, whereas genes related to TAG catabolism (ATGL and CPT I) were expressed at higher levels in the high-lipid group with the exception of ACO (*P*<0.05).

## Discussion

In the present study, the final weight and specific growth rate of fish were not significantly affected by dietary lipid levels, which were consistent with some previous studies conducted in large yellow croaker [33], Atlantic cod [34], European sea bass [35] and rainbow trout [36]. However, several studies have shown that an increase in dietary lipid can promote growth[1,2,37] or suppress growth in fish[38–41]. The growth-promoting effect of high dietary lipids was general correlated with the protein-sparing effect of dietary lipids [1,37]. Although a protein-sparing effect was reported in juvenile large yellow croaker (10 g) [25], this effect was not observed in middle-sized fish of the same species in the study of Wang *et al*. (240 g) [33] or in the present study (150 g). In contrast, growth suppression by high dietary lipids was primarily due to high-lipid-induced reduction in feed intake [40,41]. However, this phenomenon was not observed in the study of Wang *et al*. or in the present study potentially due to the high capacity of lipid tolerance in large yellow croaker[33]. Therefore, the effects of dietary lipid levels on fish growth performance are complex, which may be related to fish life stages, fish species, feed composition and feeding strategies.

The lipid content was significantly increased in the whole body and liver when dietary lipids were increased from 6 to 18%, which was supported by the findings of Wang *et al*.[33] in the same fish species. The increase of dietary lipid levels lead to higher lipid deposition has also been observed in turbot [39], Atlantic cod [34]and juvenile cobia [40]. Also, increased dietary lipid levels resulted in increased plasma TAG, NEFA and LDL-c levels, which was consistent with results previously obtained in large yellow croaker [33], grass carp [3,42] and tiger puffer [41]. Adipose tissue plays an important role in storing excessive amounts of circulating FAs in the form of TAG and in mobilizing TAG *via* breakdown into NEFAs [43]. The increased plasma NEFAs level in fish fed the high-lipid diet may be an important source of lipid accumulation in the liver of large yellow croaker. In a study on gilthead sea bream, the authors also suggested that increased adipocyte lipolysis rates in adipose tissue could be an important factor resulting in excess lipid accumulation in the liver [44]. More precisely, Donnelly *et al*.[45] reported that 59% of the TAG that accumulated in the liver of the NAFLD patients originated from NEFAs, and greater than 60% of NEFAs were from adipose tissue. Therefore, further studies are needed to determine the mechanism by which adipose tissue influences lipid homeostasis and deposition.

In the present study, increased plasma TAG level potentially indicated a higher hepatic VLDL secretion in fish fed the high-lipid diet. However, apoB100 was down-regulated as the dietary lipid content increased up to 18%, which, to some extent, may be attributable to higher n-3 LC-polyunsaturated FA (LC-PUFA) level in the high-lipid diet. Nevertheless, hepatic lipids content is a predominant driver of VLDL assembly, and insufficient lipids supplies have evidenced to promote apoB100 degradation and reduce VLDL assembly and secretion[46,47]. Moreover, plasma NEFA could be directly incorporated in to VLDL-TAG and obviously stimulate VLDL secretion [48,49]. Therefore, the increase of plasma TAG and NEFA level and hepatic lipid content in the high-lipid group suggested that hepatic VLDL secretion to peripheral tissues increased in order to reduce lipid deposition.

LPL mRNA levels were not significantly affected by dietary lipid levels in the liver, which is consistent with results reported for red sea bream [50] and large yellow croaker [33]. However, Lu *et al*. [51]reported that a high-fat diet significantly up-regulated LPL in the liver of blunt snout bream. The different fish species used in these studies potentially accounted for this disparity. Abdominal (or perivisceral) adipose tissue is the primarily lipids storage tissue in red sea bream and large yellow croaker [52], whereas the liver is the main storage tissue and stored more lipids in blunt snout bream fed a high-fat diet [51]. Even though this tissue-specific regulation of LPL by dietary lipids exists in various fish species, further studies are needed to confirm, given that LPL plays a pivotal role in LP metabolism and lipid deposition and limited researches in nutritional regulation of LPL in fish.

LDLR and LRP-1 are closely associated with LP clearance in plasma, and increased expression of these two LP receptors has been reported to improve LP clearance in hamsters [53,54]. In the present study, based on the up-regulation of LDLR and LRP-1 expression in the low- and high-lipid groups, it was supposed that plasma LP clearance was enhanced in these two groups. Among the three receptors (LDLR, LRP-1 and SRBI), the highest elevation of expression was found for the LDLR, which indicated that this receptor is vulnerable to the regulation by dietary lipids and plays a critical role in LP clearance. In mammals, loss of the LDLR leads to decreased LDL catabolism and elevated LDL levels [55]. Additionally, LDLR gene mutations result in familial hypercholesterolemia [56,57]. Compared with the control mice, *trans*-10, *cis*-12-treated C57BL/6j mice exhibit increased LDLR expression with lower plasma TAG levels [58], and LDLR^-/-^ apoB^100/100^ mice exhibit increased LRP-1 expression [11]. In the present study, SRBI expression was not significantly affected by dietary lipid content, possibly because SRBI is mainly associated with reverse cholesterol transport[59,60].

Regarding the FA transporters (CD36, FATP1 and FABPs), it was interesting that the expression of CD36, FATP1, FABP10 and FABP3 was the highest in the low-lipid group, whereas FABP11 expression was the highest in the high-lipid group. Increased expression of these genes was assumed to increased FA uptake. However, the importance and detail role of individual FA transporter may differ under various dietary lipid levels. In rats, greater transport efficiency of CD36 is found compared with FATP1 in skeletal muscle [61]. Regarding FABPs in mammals, FABP1 (homologous to fish FABP10 [62]) mainly acts as a LCFA transporter in the liver, particularly targeting ligand to β-oxidation. FABP3 is required for LCFA transport for mitochondria β-oxidation in muscle and FABP4 (homologous to fish FABP11 [62]) plays a role in TAG storage in the adipose tissue [63]. These transporters may have the similar roles in fish. Therefore, the highest expression of FABP11 in the high-lipid group could be mainly used to transport FAs to synthesis TAG, whereas FAs absorbed in the low-lipid group are potentially used for other metabolism pathways. However, these potential roles must be confirmed in further studies.

TAG synthesis and catabolism are also pivotal factors affecting lipid accumulation for a specific tissue. FAS is a key enzyme involved in *de novo* lipogenesis, and acyl-CoA:diacylglycerol acyltransferase (DGAT) catalyzes the final and only committed step in the biosynthesis of TAG [43]. ATGL has been proven to catalyze the initial step of TAG hydrolysis [64,65], and CTP1 and ACO both catalyze the rate-limiting step in FA β-oxidation. In the present study, hepatic gene expression related to TAG synthesis (DGAT2) and FA oxidation (CPT I and ACO) was attenuated in the high-lipid group, which was supported by results previously obtained in blunt snout bream [5] and large yellow croaker [33]. Reduced DGAT2 expression could be partially due to a feedback mechanism involving excessive lipid accumulation in the liver, which has been observed in a mouse model of high-fat diet-induced obesity [66–68]. Furthermore, the inhibitory effect of lipogenesis by increased n-3 LC-PUFA level in the high-lipid diet could be another factor involved in the reduction of DGAT2 expression [69,70]. However, n-3 LC-PUFA could lead to the unexpected down-regulation of CPT I and ACO expression in the high-lipid group. Lu *et al*. [5] reported that high levels of n-3 LC-PUFA significantly altered the hepatic mitochondrial membrane FA composition and CPT I kinetics in blunt snout bream, and the expression of hepatic CPT I and ACO was also down-regulated. Similar results have also been obtained in rats [71]. Furthermore, this decreased FA oxidation activity could result in increased lipid deposition in the liver of large yellow croaker. Increased hepatic FAS expression was observed in the low-lipid group compared with the control group, which was potentially due to the elevation of *de novo* lipogenesis in response to the excess carbohydrates in the low-lipid diet [72]. However, the *de novo* lipogenesis process is quite limited in fish [72]. Therefore, under the condition of reduced availability of FA sources, DGAT2 expression in the low-lipid group reduced compared with the control group and accompanied by a lower lipid content in the liver. In addition, the down-regulation of ATGL and CPT I expression potentially correlated with higher carbohydrate and lower lipid levels in the low-lipid group compared with the control group because that the provision of digestible carbohydrates in diets could spare the use of lipids as sources of energy [72,73].

## Conclusions

Results of the present study demonstrated that different dietary lipid levels could regulate various metabolic pathways to affect hepatic lipid deposition in large yellow croaker at the transcriptional level. In fish fed the high-lipid diet, increased lipoprotein clearance and fatty acid uptake and decreased fatty acid β-oxidation were potentially involved in the increased hepatic lipid deposition. For fish fed the low-lipid diet, to some extent, lipoprotein clearance together with fatty acid uptake and *de novo* synthesis increased, whereas triacylglycerol catabolism decreased. However, lipid accumulation in the liver decreased, primarily due to low dietary lipid intake in fish fed the low-lipid diet.
